# Comparative Outcomes of the Next-Generation Extended Depth-of-Focus Intraocular Lens and Enhanced Monofocal Intraocular Lens in Cataract Surgery

**DOI:** 10.3390/jcm14144967

**Published:** 2025-07-14

**Authors:** Do Young Kim, Ella Seo Yeon Park, Hyunjin Park, Bo Yi Kim, Ikhyun Jun, Kyoung Yul Seo, Ahmed Elsheikh, Tae-im Kim

**Affiliations:** 1 Yonsei University College of Medicine, Seoul 03722, Republic of Korea; dyaiden@naver.com; 2 Department of Ophthalmology, Institute of Vision Research, Yonsei University College of Medicine, Seoul 03722, Republic of Korea; kpark1107@gmail.com (E.S.Y.P.); hyunjinp6470@gmail.com (H.P.); hadesdual@yuhs.ac (I.J.); seoky@yuhs.ac (K.Y.S.); 3 Department of Refractive Surgery, B&VIIT Eye Center, Seoul 06615, Republic of Korea; boyikim@gmail.com; 4 School of Engineering, University of Liverpool, Liverpool L69 7ZX, UK; elsheikh@liverpool.ac.uk; 5 Beijing Advanced Innovation Centre for Biomedical Engineering, Beihang University, Beijing 100191, China; 6 National Institute for Health Research (NIHR) Biomedical Research Centre for Ophthalmology, Moorfields Eye Hospital NHS Foundation Trust and UCL Institute of Ophthalmology, London EC1V 2PD, UK; 7 Department of Ophthalmology, Corneal Dystrophy Research Institute, Yonsei University College of Medicine, Seoul 03722, Republic of Korea; 8 Department of Material Science and Engineering, Yonsei University, Seoul 03722, Republic of Korea; 9 Affilate Faculty, Material Research Center for Batteries, Pohang University of Science and Technology (POSTECH), Pohang 37673, Republic of Korea

**Keywords:** extended depth-of-focus (EDOF) intraocular lenses (IOLs), cataract surgery IOLs, presbyopia correction, clinical efficacy

## Abstract

**Background/Objectives:** A new, purely refractive extended depth-of-focus (EDOF) intraocular lens (IOL) was designed with a continuous change in power to bridge the gap between monofocal and multifocal IOLs. This study aimed to evaluate the real-world clinical outcomes of the new EDOF IOL compared with those of the enhanced monofocal IOL. **Methods:** A retrospective analysis was conducted on 100 eyes from 50 patients undergoing bilateral cataract surgery with either the PureSee™ EDOF (ZEN00V) or Eyhance™ (ICB00) monofocal IOL at a single institution. Visual acuity, defocus curves, contrast sensitivity, and patient-reported outcomes were evaluated three months postoperatively. **Results:** The ZEN00V group demonstrated superior uncorrected intermediate (0.11 ± 0.08 vs. 0.17 ± 0.11 logMAR, *p* = 0.006) and near visual acuity (0.25 ± 0.08 vs. 0.31 ± 0.13 logMAR, *p* = 0.023) compared to the ICB00 group, with comparable distance visual acuity. Both groups exhibited comparable defocus curves and contrast sensitivity. While photic phenomena were more frequent in the ZEN00V group, spectacle dependence was significantly lower for near vision (36% vs. 80%, *p* = 0.002) and comparable for intermediate and far vision. **Conclusions:** The PureSee™ EDOF IOL demonstrated enhanced intermediate and near vision with minimal compromise to distance vision while maintaining high contrast sensitivity. It also offered significant spectacle independence and patient satisfaction, making it a promising option for presbyopia correction.

## 1. Introduction

The clinical outcomes of cataract surgery have significantly improved due to advancements in preoperative biometric measurement techniques, improvements in intraocular lens (IOL) power calculation formulas, development of novel surgical techniques, and introduction of high-performance IOLs [1,2,3,4]. Monofocal IOLs are the most commonly implanted intraocular lenses in cataract surgery due to their excellent outcomes for single-focus vision, low incidence of photic phenomena, and relatively low cost. However, as monofocal lenses provide optimal uncorrected visual acuity at a fixed distance, additional spectacles are required to enhance the quality of visual performance in daily activities [3,5]. To address this limitation, presbyopia-correcting lenses have been introduced to the market, aiming to further promote greater spectacle independence.

Multifocal IOLs, designed based on simultaneous vision, have been developed to improve intermediate and near vision. However, compared with monofocal IOLs, both refractive and diffractive designs inevitably involve some degree of light dispersion, reducing contrast sensitivity and increasing visual disturbances, such as photic phenomena. These factors contribute to dissatisfaction with multifocal IOLs despite their ability to provide an enhanced range of vision and greater spectacle independence [6,7,8]. Postoperative residual refractive error is one of the major causes of decreased postoperative visual quality in patients with multifocal IOLs. Although advancements in IOL power prediction have improved accuracy, multifocal IOL implantation remains limited in cases where accurate power calculation is challenging, such as in short eyes, highly myopic eyes, or those with a history of refractive surgery [9,10].

To address these challenges, new technologies have emerged to improve IOL performance while minimizing the unwanted photic phenomena commonly associated with multifocal IOLs. Extended depth-of-focus (EDOF) IOLs have been developed to bridge the gap between monofocal and multifocal IOLs. EDOF IOLs provide a more seamless and continuous range of focus across intermediate to far distances, elongating the focal points to offer an enhanced and continuous range of vision [3,11,12]. They provide marginally improved intermediate vision with photic phenomena comparable to conventional monofocals [13].

A next-generation EDOF IOL, TECNIS PureSee™ (Johnson and Johnson Surgical Vision, Irvine, CA, USA), has recently been developed. The lens features a purely refractive EDOF design with a continuous change in the power profile, maintaining vision quality and contrast sensitivity comparable to those of monofocal IOLs. A few studies have reported optical bench or clinical outcomes with the TECNIS PureSee™ IOL. Both preclinical and randomized clinical studies demonstrated improved intermediate and near visual performance compared to monofocal IOLs. Distance vision and photic phenomena remained comparable to those of monofocal IOL [11,14,15]. However, visual performance and patient satisfaction results in real-world clinical settings remain largely unknown. This study aimed to evaluate the real-world clinical outcomes following the implantation of this next-generation EDOF IOL, including distance, intermediate, and near vision, contrast sensitivity, and defocus curve data. These potential advantages will be assessed relative to the post-surgery performance of the monofocal Eyhance™ IOLs, which also offers a slight extension of depth of focus.

## 2. Materials and Methods

### 2.1. Study Design and Participants

This single-center, respective study was conducted in the Severance Eye Hospital, Yonsei University College of medicine, Seoul, Korea. We retrospectively reviewed the medical charts and clinical data of patients who underwent cataract surgery between June 2021 and May 2024. Study participants were eligible for inclusion if they were over 40 years old and presented with preoperative astigmatism ≤ 1.0 diopter (D). Patients were excluded if they were over 85 years of age, had an axial length > 26.0 mm or <22.5 mm, a history of prior ocular surgery or trauma, ocular abnormalities, or diseases other than cataracts that could affect postoperative visual acuity. Patients with an axial length of >26.0 mm were excluded based on prior studies reporting an association between absolute prediction error and elongated axial length [16,17]. Patients with intra- or post-operative complications were also excluded. One-to-one (1:1) case matching was performed, and the control eyes were matched for age (±5 years).

### 2.2. Surgical Technique

All surgeries were performed by a single surgeon (Tae-Im Kim) at Severance Eye Hospital in Seoul, South Korea. All procedures were performed under topical anesthesia with 0.5% proparacaine hydrochloride. A main incision was made on the axis of the steepest corneal curvature, and a 20 G paracentesis was performed. Conventional phacoemulsification, along with irrigation, aspiration, and capsule polishing, was performed using the Centurion Vision System (Alcon Laboratories, Inc., Forth Worth, TX, USA). Finally, the IOL was implanted in the capsular bag, and all incisions were sealed using stromal hydration.

### 2.3. IOLs

In this study, the TECNIS PureSee™ EDOF IOL (Model ZEN00V) and TECNIS Eyhance™ enhanced monofocal IOL (Model ICB00) were used. The ZEN00V is a next-generation refractive EDOF lens comprising a one-piece soft acrylic aspheric foldable posterior chamber lens with a total diameter of 13.0 mm and an optical diameter of 6.0 mm. It features a biconvex, wavefront-designed anterior aspheric surface to compensate for the average corneal spherical aberration and a posterior refractive surface that provides a continuous power change to achieve an extended depth of focus. The ICB00, a one-piece soft acrylic hydrophobic foldable posterior chamber lens, shares the same overall geometry and dimensions as the ZEN00V. Its higher-order aspheric anterior surface provides a continuous change in power from the periphery to the center, slightly extending the depth of focus. Both lenses have an optical A-constant of 119.3 and are available in dioptric powers ranging from +5.0 D to +34.0 D, in 0.5 D increments.

### 2.4. Measurements

All participants underwent comprehensive ophthalmic examinations before the study, including screening for ocular and systemic diseases, visual acuity assessment, manifest refraction, intraocular pressure measurement, keratometry, slit-lamp examination, dilated fundoscopy, specular microscopy, and optical biometry.

Visual acuity was assessed under photopic conditions (85 candelas/square meter (cd/m^2^)) with 100% contrast. Monocular corrected distance visual acuity (CDVA) and uncorrected distance visual acuity (UDVA) were measured at 4 months using an Early Treatment Diabetic Retinopathy Study (ETDRS) chart (Precision Vision, Woodstock, IL, USA). Monocular uncorrected intermediate visual acuity (UIVA at 66 cm) and uncorrected near visual acuity (UNVA at 40 cm) were assessed using handheld ETDRS vision cards (Precision Vision, Woodstock, IL, USA). Visual acuity measurements were converted to the logarithm of the minimum angle of resolution (logMAR) for statistical analysis. Manifest refraction was determined using standard optometric methods. Keratometry was performed using a Topcon KR 8800 auto-kerato-refractometer (Topcon Corporation, Tokyo, Japan), while optical biometry and specular microscopy were performed using the IOL Master 700 (Carl Zeiss Meditec AG, Jena, Germany) and EM-4000 (Tomey GmbH, Nuremberg, Germany), respectively. The power of the implanted IOL was calculated by using the Barrett Universal II formula. No individualized adjustment was made to the IOL constants. The IOL power was selected to achieve a postoperative refraction of −0.25D, favoring slight myopia.

### 2.5. Postoperative Outcome Measures

Following cataract surgery, patients were examined one day, one week, one month, and three months postoperatively. At three months, all patients underwent the following examinations:

*Visual acuity*. Monocular CDVA and UDVA were assessed using the same methods as in the preoperative examinations. Monocular UIVA (at 66 cm) and UNVA (at 40 cm) were measured using handheld ETDRS vision cards (Precision Vision, Woodstock, IL, USA).

*Defocus curve*. Binocular defocus curves were obtained by defocusing the eyes to +1.50 D spherical from the manifest refraction value. The same trial frame used to measure BCVA was utilized for defocus curve measurement to minimize the effect of vertex distance. Negative spherical trial lenses were added in increments of −0.50 D to −4.00 D. Binocular visual acuity was measured at each increment using the ETDRS chart at 4 m.

*Contrast sensitivity*. Contrast sensitivity was measured under photopic (target luminance: 85 cd/m^2^) and mesopic (target luminance: 3 cd/m^2^) conditions using subjective refraction. Sensitivity was measured at five spatial frequencies (1.5, 3, 6, 12, and 18 cycles per degree). The functional acuity contrast test (F.A.C.T) was performed using the Optec 6500 view-in test system (Stereo Optical Co., Inc., Chicago, IL, USA). The F.A.C.T. was conducted under consistent conditions, with control limited to mesopic and photopic lightening conditions.

*Questionnaire.* Patients completed a questionnaire assessing postoperative photic phenomena (halo, glare, and starburst), spectacle independence during daily tasks (distance, intermediate, and near vision), and overall satisfaction with outcomes. Participants also indicated whether they would recommend the same IOL to others. Survey responses were recorded as ‘yes’ or ‘no’. A detailed version of the questionnaire provided to the participants is provided as Supplementary Material (Table S1).

### 2.6. Statistical Analysis

A sample size of 42 subjects was required to achieve 80% power to detect a 0.10 logMAR or greater difference in mean VA between the two groups for UIVA and UNVA. Standard deviation of 0.16 logMAR was assumed, and a two-sample *t*-test at a one-sided alpha of 0.025 was used. Our study included more than 42 eyes for each group.

Baseline characteristics are presented as mean ± standard deviation for continuous variables and numbers with percentages for categorical variables. Missing data were not imputed. Differences between measurement indicators were analyzed using Pearson’s chi-square test for categorical variables. However, when the expected frequency in any cell was less than 5, Fisher’s exact test was applied instead. Differences between measurement indicators were analyzed using Student’s independent *t*-test for continuous variables. To adjust for inter-eye correlation in monocular measurements, the generalized estimating equation (GEE) with an exchangeable correlation structure was used for group comparison. All tests were two-tailed, and a *p*-value < 0.05 was considered statistically significant. Statistical analyses were performed using SPSS software (version 22.0; IBM Corp., Armonk, NY, USA) and R version 4.3.3 (R Foundation for Statistical Computing, Vienna, Austria).

## 3. Results

A total of 58 eyes from 29 patients for the ZEN00V group and 68 eyes from 34 patients for the ICB00 group, with no missing medical records or loss to follow-up before the 3-month period, were evaluated. Four patients from the ZEN00V group and six patients from the ICB00 group were excluded because of intra- or post-operative complications.

A total of 100 eyes from 50 patients who underwent cataract surgery using the ZEN00V IOL (*n* = 50) and ICB00 IOL (*n* = 50) in both eyes were matched and evaluated in the current study. Table 1 presents the baseline demographic and preoperative ophthalmic measurements of participants in both groups.

Patient demographics were comparable between the groups. The mean (±SD) age was 69.48 ± 5.83 years in the ZEN00V group and 72.44 ± 5.80 years in the ICB00 group (*p* = 0.30). The preoperative spherical equivalent (SE) was 0.88 ± 1.62 in the ZEN00V group and 0.65 ± 1.63 in the ICB00 group (*p* = 0.68). Both groups included a higher proportion of female patients (*p* = 0.06). Preoperative uncorrected visual acuity (0.38 ± 0.29 vs. 0.48 ± 0.34, *p* = 0.20) and best-corrected visual acuity (0.15 ± 0.19 vs. 0.23 ± 0.27, *p* = 0.13) were comparable between the groups. No intra- or post-operative complications, such as endophthalmitis, secondary glaucoma, or cystoid macular edema, were reported. All participants included in the analysis were followed up for three months after cataract surgery.

The refractive and visual outcomes at three months postoperatively are shown in Table 2. The postoperative SE was −0.46 ± 0.60D in the ZEN00V group and −0.28 ± 0.27 D in the ICB00 group (*p* = 0.09). Both groups achieved fine results in the UDVA (0.08 ± 0.10 vs. 0.10 ± 0.11, *p* = 0.53) and CDVA (0.01 ± 0.03 vs. 0.01 ± 0.02, *p* = 0.21) without significant differences.

However, monocular UIVA at 66 cm was 0.11 ± 0.08 logMAR in the ZEN00V group compared to 0.17 ± 0.11 logMAR in the ICB00 group (*p* = 0.006). The mean UNVA at 40 cm was 0.25 ± 0.07 logMAR in the ZEN00V group compared to 0.31 ± 0.13 logMAR in the ICB00 group (*p* = 0.023). The ZEN00V group also showed consistent and superior outcomes in actual clinical settings. For UIVA, 72% (36/50) of the ZEN00V group achieved monocular UIVA better than 0.20 logMAR, compared to 50% (25/50) of the ICB00 group. For UNVA, 14% (7/50) of the ZEN00V group achieved a UNVA better than 0.20 logMAR, compared to 10% (5/50) of the ICB00 group.

**Figure 1:**
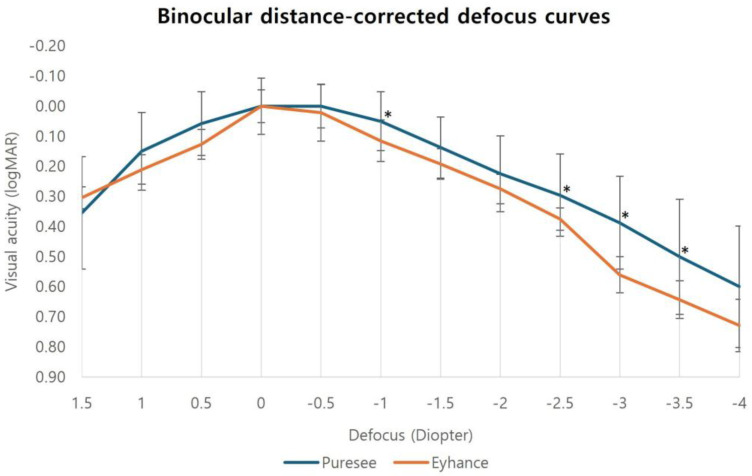
Binocular defocus curves obtained from the Puresee (ZEN00V) and Eyhance (ICB00) groups. Defocused vision with the ZEN00V lens was significantly higher at −1.0 D and in the range of −2.50 D to −3.50 D compared to the ICB00 lens. D, diopters; LogMAR, logarithm of the minimal angle of resolution. * *p* < 0.05.

The defocus curves for each IOL group are shown in Figure 1. Both curves had a U-shape with a peak at 0.00 D, corresponding to maximum vision. The defocus curve of the ZEN00V lens generally outperformed that of the ICB00 lens, except at +1.5 D, although this difference was not statistically significant. Direct comparison of defocus curves revealed that defocused vision with the ZEN00V lens was significantly higher at −1.0 D and in the range of −2.50 D to −3.50 D compared to the ICB00 lens. The binocular defocus curve of the ZEN00V lens showed a mean visual acuity better than 0.20 logMAR within the +1.00 D to –1.50 D interval.

**Figure 2:**
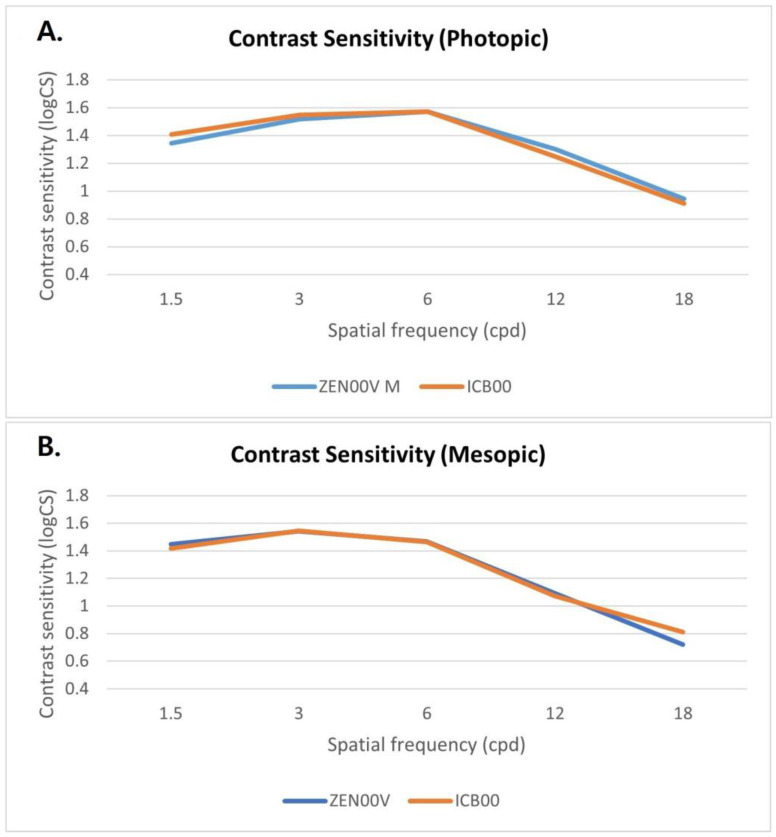
Monocular contrast sensitivity values under photopic (**A**) and mesopic (**B**) conditions without glare. No significant differences were observed between groups across spatial frequencies (all *p* > 0.05). logCS, log contrast sensitivity; cpd, cycles per degree.

The mean contrast sensitivity values under both photopic and mesopic conditions without glare are shown in Figure 2. No significant differences were observed between groups across spatial frequencies (all *p* > 0.05).

Photic phenomena were reported more frequently in the ZEN00V group than in the ICB00 group. In the ZEN00V group, four patients (16%) reported halos, four (16%) reported glare, and one (4%) reported starbursts. In contrast, two patients (8%) in the ICB00 group reported halos, with no other visual symptoms noted (all *p* > 0.05). None of the patients in either group reported the need for far distance correction (*p* > 0.05). One patient in the ZEN00V group and one in the ICB00 group reported a need for intermediate correction (*p* > 0.05). For near vision correction, 20 patients (20%) in the ICB00 group required spectacles, compared to only 9 (36%) in the ZEN00V group (*p* = 0.002). Both groups demonstrated over 90% satisfaction rates. Additionally, 100% of patients in both groups indicated they would recommend the same type of IOL to others (Table 3).

## 4. Discussion

This study compared the TECNIS Puresee™ ZEN00V, a novel refractive EDOF IOL, to the TECNIS Eyhance™ ICB00, an enhanced monofocal IOL from the same platform. The results demonstrated that the new refractive EDOF IOL extended the range of vision while preserving distance visual acuity comparable to that of the enhanced monofocal IOL. Patients who implanted the refractive EDOF IOL achieved significantly better intermediate and near visual acuities compared to those who implanted the enhanced monofocal IOL. Additionally, the new refractive EDOF IOL generally outperformed the enhanced monofocal IOL in the binocular defocus test. Contrast sensitivity was comparable between the two groups across all spatial frequencies under both mesopic and photopic conditions.

A previous multicenter, double-blind, randomized clinical trial (RCT) and the optical bench analysis of the new refractive EDOF IOL corroborated the findings of this study [11]. In a previous study, monocular CDVA was comparable between the refractive EDOF IOL and enhanced monofocal IOL. Intermediate visual acuity was 0.13 ± 0.08 for the refractive EDOF IOL and 0.18 ± 0.14 for the enhanced monofocal IOL, demonstrating a significant improvement of 0.05 logMAR in our study (*p* = 0.0127). Near visual acuity was 0.37 ± 0.10 logMAR for the refractive EDOF IOL and 0.43 ± 0.16 for the enhanced monofocal IOL (*p* = 0.0137) [11]. Previous studies on the enhanced monofocal IOL reported monocular intermediate visual acuity values ranging from 0.16 to 0.30 logMAR and near visual acuity values ranging from 0.32 to 0.46 logMAR [18,19,20,21]. The visual performance of the enhanced monofocal IOL generally aligns well with the findings of both the aforementioned RCT and this study.

The differences in intermediate and near visual acuity between the new refractive EDOF IOL and the enhanced monofocal IOL in this study showed good correlation with previous reports. Although statistically not significant, postoperative SE was more myopic in the new refractive EDOF IOL group, which may have contributed to the superior intermediate and near visual acuity outcomes observed in this group.

The defocus curves for both the new refractive EDOF IOL and the enhanced monofocal IOL showed a single-focus profile extending to intermediate vision, with a peak corresponding to the best visual acuity at 0.00 D. The defocus curve for the new refractive EDOF IOL showed an overall superior trend compared with that of the enhanced monofocal IOL. Specifically, the new refractive EDOF IOL demonstrated a <0.10 logMAR range between +0.50 D and −1.00 D and a <0.20 logMAR range between +1.00 D and −1.50 D. Preclinical optical bench simulations indicated that the new refractive EDOF IOL offered a range of vision comparable to that of diffractive EDF IOLs while exhibiting greater tolerance to refractive error [15]. In a previous RCT, monocular distance-corrected defocus curves were acquired from +1.00 D to −2.50 D. The new refractive EDOF IOL demonstrated a more extended range of vision than the enhanced monofocal IOL [11].

A follow-up article by Black et al. further highlighted that the new refractive EDOF IOL demonstrated high-quality distance vision comparable to monofocal IOL, even in the presence of residual refractive error [14]. Since it is not possible to completely control all variables, predicting postoperative residual refractive errors and pseudo-accommodation following cataract surgery remains an ongoing challenge [13]. Residual refractive errors can compromise visual performance, particularly in multifocal IOLs [3,8]. An EDOF IOL may be a viable option, as it is more tolerant of residual refractive errors and provides an extended range of vision without a definite compromise in visual acuity compared to monofocal IOLs [14].

Aside from the studies mentioned above, Alfonso-Bartolozzi et al. reported a single-centered retrospective study on visual outcome of the new refractive EDOF IOL using clinical data [22]. Alfonso-Bartolozzi et al. demonstrated that the mean postoperative monocular UDVA and corrected distance VA at three months postoperatively were 0.10 ± 0.15 and 0.05 ± 0.09 logMAR, respectively. A monocular defocus curve demonstrated a visual acuity below 0.20 logMAR across the vergence range from 0.00 D to −2.00 D [22]. The visual performance generally correlated well with our study results.

The contrast sensitivity test correlates better with identifying real-world objects and provides a more accurate measure of visual sensitivity and quality than visual acuity alone because it evaluates spatial vision performance in both size and contrast [23,24]. Some studies have demonstrated that multifocal IOLs and EDOF IOLs may be associated with a decrease in contrast sensitivity [3,8]. Multifocal IOLs project simultaneous images onto the retina, causing overlapping of near and far images. This enables vision at various distances but simultaneously conveys blurred images to the retina, thereby compromising vision quality. EDOF IOLs offer a continuous range of focus without distinct asymmetry in IOL power distribution, minimizing the occurrence of secondary out-of-focus images [8,25,26].

The new refractive EDOF IOL evaluated in this study, TECNIS Puresee™ EDOF IOL, was designed to reduce photic phenomena. A continuous change in refractive power, achieved through a gradual change in the curvature of the posterior surface of the lens, eliminates sources of scatter. In our study, mesopic and photopic contrast sensitivity without glare were comparable between the two IOL groups across all spatial frequency ranges. A previous clinical study similarly showed no compromise in contrast sensitivity under mesopic conditions across all spatial frequency ranges [11,14]. The results of this study align with those of previous clinical trials demonstrating high-quality distance vision provided by the new refractive EDOF IOL.

Regarding the dysphotopsia profile obtained from patient questionnaires, less than 20% of patients in the new refractive EDOF IOL group reported photic phenomena. However, the incidence of photic phenomena at three months postoperatively was higher in the new refractive EDOF IOL group than in the enhanced monofocal IOL group. Optical bench and simulation studies have shown a dysphotopsia profile comparable to that of monofocal IOL in the presence of refractive errors [15]. In a previous clinical study, most patients reported no experience of or minimal bother from halos (91.7%), stardusts (95.0%), and glare (95.0%). While most participants did not experience such symptoms, photic phenomena were observed more frequently with the new refractive EDOF IOL [11]. This monofocal-like dysphotopsia profile of the new refractive IOL may be explained by the smooth change in curvature of the posterior surface of the lens, as shown in a previous simulation [15].

A higher proportion of patients in the new refractive EDOF IOL group reported spectacle independence for intermediate and near vision compared with the enhanced monofocal IOL group, consistent with visual acuity and defocus curve results. Spectacle independence in a prior study was also reported to be 100% for the new refractive EDOF IOL, which aligns with the findings of our study [14]. Other studies using enhanced monofocal IOL have also reported 100% spectacle independence for intermediate vision and moderate independence for near vision following enhanced monofocal IOL implantation [27].

The demand for spectacle-free intermediate and near vision for daily activities is increasing, driven by longer life expectancy and a greater emphasis on quality of life [25,28]. As presbyopia prevalence increases with age, the number of individuals affected is expected to peak at approximately 2.1 billion by 2030 [28]. In such context, the new refractive EDOF IOL is expected to provide distance vision comparable to that of monofocal IOLs while extending the depth of focus to offer a broader range of vision. In this study, UDVA was comparable between the two groups, although the mean postoperative SE was more myopic in the new refractive EDOF IOL group than in the enhanced monofocal IOL group. This finding suggests that the new refractive EDOF IOL tolerates a degree of postoperative residual refractive error, likely enhancing patient satisfaction after surgery.

This study had some limitations. First, it was a single-center study with a small sample size confined to Koreans. Further studies with larger sample sizes or involving multiple centers are necessary to validate these findings. Additionally, it has inherent limitations due to its retrospective design. The quality of vision was evaluated subjectively using a questionnaire in the current study. Future investigations involving objective indicators, such as optical aberrations, would provide more comprehensive evaluation of the new refractive EDOF IOL. Nonetheless, the current study did compare tolerance to refractive error between the two groups through the binocular defocus curve. Moreover, reading speed and stereopsis were not sufficiently evaluated in this study. It would be of interest to assess the aforementioned factors postoperatively in patients implanting new refractive EDOF IOLs.

## 5. Conclusions

This study evaluated visual performance at different distances, patient satisfaction, defocus curves, and contrast sensitivity following cataract surgery using the new refractive EDOF TECNIS Puresee™ IOL. Compared with the enhanced monofocal IOL, the new refractive IOL demonstrated improved intermediate and near vision, while maintaining comparable distance vision or contrast sensitivity. The new refractive IOL also provided significant spectacle independence and patient satisfaction, making it a promising option for presbyopia correction. Additionally, our real-world clinical data demonstrated the reproducibility of findings from preclinical simulations and the RCT. Therefore, the TECNIS Puresee™ IOL is expected to be a valuable option for cataract surgeons, offering excellent distance and intermediate vision and functional near vision with acceptable photic phenomena.

## Figures and Tables

**Table 1:** Preoperative patient demographics.

Baseline Characteristics	Puresee ZEN00V	Eyhance ICB00	*p*-Value
Patients/eyes (*n*)	25/50	25/50	
Age (years)	69.48 ± 5.83	72.44 ± 5.80	0.30
Sex, *n* (%)			0.06
Male	5 (20%)	9 (36%)	
Female	20 (80%)	16 (64%)	
Visual acuity (LogMAR)			
UDVA	0.38 ± 0.29	0.48 ± 0.34	0.20
BCVA	0.15 ± 0.19	0.23 ± 0.27	0.13
Spherical equivalent (D)	0.88 ± 1.62	0.65 ± 1.63	0.68
K values			
K1 (D)	43.66 ± 1.11	43.63 ± 1.63	0.93
K2 (D)	44.21 ± 1.11	44.33 ± 1.55	0.75
Axis (°)	86.94 ± 46.09	96.54 ± 60.44	0.42
Axis length (mm)	23.54 ± 0.66	23.61 ± 0.83	0.74

Values are presented as mean ± standard deviation. LogMAR, logarithm of the minimal angle of resolution; D, diopters.

**Table 2:** Visual acuity at postoperative three months.

	Puresee ZEN00V	Eyhance ICB00	*p*-Value
UDVA (logMAR)	0.08 ± 0.10	0.10 ± 0.11	0.53
BCVA (logMAR)	0.01 ± 0.03	0.01 ± 0.02	0.21
UIVA (logMAR)	**0.11 ± 0.08**	**0.17** **±** **0.11**	**0.006** **^†^**
UNVA (logMAR)	**0.25 ± 0.07**	**0.31** **±** **0.13**	**0.023** **^†^**
Spherical equivalent (D)	−0.46 ± 0.60	−0.28 ± 0.27	0.09

Values are presented as mean ± standard deviation. LogMAR, logarithm of the minimal angle of resolution; D, diopters; UDVA, uncorrected distance visual acuity; DCDVA, distance-corrected distance visual acuity; UIVA, uncorrected intermediate visual acuity; UNVA, uncorrected near visual acuity. ^†^ *p* < 0.01. Significant values are indicated in bold font.

**Table 3:** Results for the patient questionnaire regarding photic phenomena, spectacle dependence, overall satisfaction, and recommendations for each IOL.

	Puresee (*n* = 25)	Eyhance (*n* = 25)	*p*-Value
**Photic phenomena**			
Halo	4 (16%)	2 (8%)	0.66
Glare	4 (16%)	0	0.11
Starburst	1 (4%)	0	1.00
**Spectacle dependence**			
Distance	0	0	1.00
Intermediate	1 (4%)	1 (4%)	1.00
Near	9 (36%)	20 (80%)	**0.002 ^†^**
**Overall satisfaction**	24 (96%)	23 (92%)	1.00
**Recommendation**	25 (100%)	25 (100%)	1.00

Values are presented as frequency (percentage). ^†^ *p* < 0.01. Significant values are indicated in bold font.

## Data Availability

The datasets used during the current study are available from the corresponding author upon reasonable request. All data generated or analyzed during this study are included in this published article.

## Supplementary Materials

The following supporting information can be downloaded at: https://www.mdpi.com/article/10.3390/jcm14144967/s1. Table S1: Patient questionnaire regarding visual symptoms, spectacle dependence, and overall satisfaction. Figure S1: Image retrieved from article by Chang D [29].

## Abbreviations

The following abbreviations are used in this manuscript:

CDVA	Corrected distance visual acuity
EDOF	Extended depth of focus
IOL	Intraocular lens
LogMAR	Logarithm of the minimum angle of resolution
RCT	Randomized clinical trial
SE	Spherical equivalent
UDVA	Uncorrected distance visual acuity
UIVA	Uncorrected intermediate visual acuity
UNVA	Uncorrected near visual acuity
