# Supplementary material for: Comparative Outcomes of the Next-Generation Extended Depth-of-Focus Intraocular Lens and Enhanced Monofocal Intraocular Lens in Cataract Surgery
Source: J Clin Med. 2025 Jul 14;14(14):4967. doi: 10.3390/jcm14144967 (PMC12295884; doi:10.3390/jcm14144967)
Supplement: Supplementary file 1 [file jcm-14-04967-s001.zip › jcm-3708275-supplementary.pdf]

SUPPLEMENTARY MATERIAL

Table S1. Patient questionnaire regarding visual symptoms, spectacle dependence and overall satisfaction.

**Question**	**Answer**
1. Do you experience discomfort in your daily life due to halo?	Yes / No
2. Do you experience discomfort in your daily life due to glare?	Yes / No
3. Do you experience discomfort in your daily life due to starburst?	Yes / No
4. Do you need spectacles to perform everyday activities at distance vision? (ex. TV)	Yes / No
5. Do you need spectacles to perform everyday activities at intermediate vision? (ex. Computer)	Yes / No
6. Do you need spectacles to perform everyday activities at near vision? (ex. Book)	Yes / No
7. Are you satisfied with the outcomes of cataract surgery using Eyhance intraocular lens?	Yes / No
8. Would you recommend cataract surgery using Eyhance intraocular lens to your friends or relatives?	Yes / No

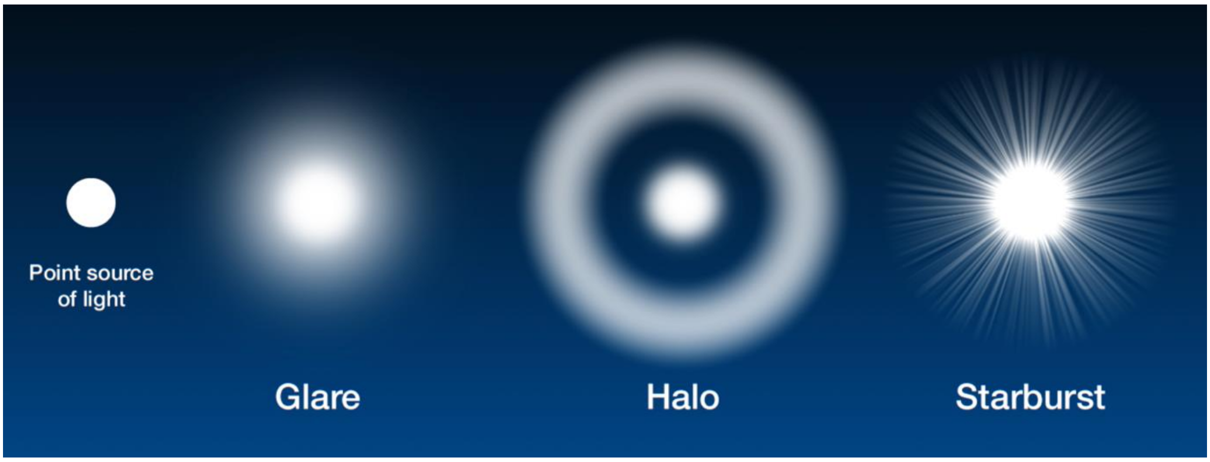

Figure S1. Image citation : Chang, Daniel. (2016). “Figure 1 : There are three distinct types of photopsias, or distortions of a point source of light.” Night Vision and Presbyopia-Correcting IOLs. Millennial Eye, Jul/Aug 2016. <https://millennialeye.com/articles/2016-jul-aug/night-vision-and-presbyopia-correcting-iols/>.
